# Outbreak of Brazilian Spotted Fever, Southeastern Brazil, 2018

**DOI:** 10.3201/eid3209.260548

**Published:** 2026-09

**Authors:** Jardel Brasil, Rodrigo N. Angerami, Maria R. Donalisio

**Affiliations:** Municipal Health Department, Americana, Brazil (J. Brasil); State University of Campinas, Campinas, Brazil (J. Brasil, R.N. Angerami, M.R. Donalisio)

**Keywords:** Brazilian spotted fever, Rickettsia rickettsii, Amblyomma sculptum, ticks, tick-borne, vector-borne infections, bacteria, bacterial infections, outbreaks, public health, Brazil

## Abstract

Brazilian spotted fever is a severe tickborne rickettsial disease endemic to Brazil. We report the clinical and epidemiological characteristics of an outbreak of 15 cases of Brazilian spotted fever caused by *Rickettsia rickettsii* in 2018 with high lethality. Our report highlights the need for early diagnosis to avert deaths.

Brazilian spotted fever (BSF) is caused by *Rickettsia rickettsii* bacteria and transmitted in endemic areas of the interior of São Paulo state, southeastern Brazil, by *Amblyomma sculptum* ticks. Transmission typically occurs in forested areas along rivers and streams inhabited by capybaras, which are the main host for the tick vector and an amplifier host for *Rickettsiae* (*1*–*3*). We describe a BSF outbreak characterized by a high number of confirmed cases, specific circumstances of transmission, and associated deaths.

An eco-epidemiologic investigation in 2018 in the municipality of Americana, Brazil, identified a cluster of 15 laboratory-confirmed cases of BSF in a periurban area (*3*) (Appendix Figure). The outbreak lasted 43 days, from April 26, 2018, the date of symptom onset of the index case, to June 7, 2018, when symptom onset occurred in the last reported case (Figure). We applied Brazilian Ministry of Health criteria to confirm each case (*4*) (Appendix Table 1). This study was approved by the Research Ethics Committee of the Faculty of Medical Sciences of the State University of Campinas (protocol no. 5,474,734).

**Figure:**
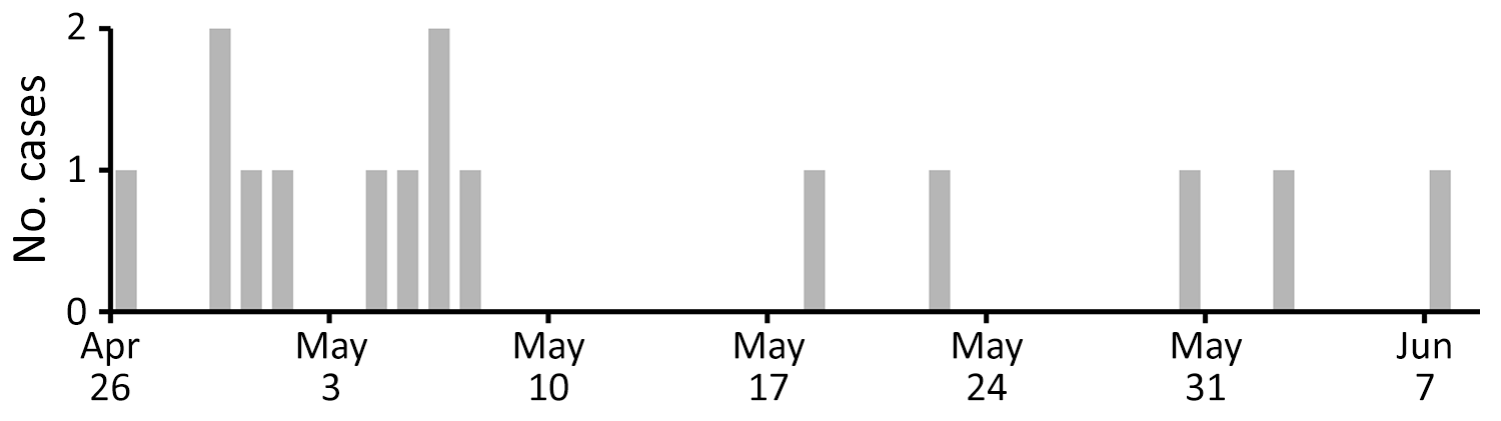
Temporal distribution of Brazilian spotted fever cases, by date of onset of symptoms, during outbreak in Americana, Brazil, 2018.

We noted the most prevalent symptoms in each case included fever, headache, and myalgia. We also observed an increased frequency in symptom severity, including respiratory failure, renal dysfunction, jaundice, neurologic symptoms, shock, and hemodynamic instability at the time of suspicion (Appendix Table 2). There were 11 deaths out of 15 cases. Of note, severe febrile-icteric and hemorrhagic syndromes were prevalent.

The case-patients’ mean age was 37 years (range 2–65 years); 14 were male and 1 female, and 6 were persons of color (Table). The case-patients had a median of 3 (range 1–4) medical consultations before hospitalization, indicating diagnostic delay. The 12 patients who were hospitalized had a median of 4 (range 2–11) days from symptom onset to hospitalization. Among the hospitalized patients who survived, the length of hospital stay was 10–14 (median 12) days. Among patients with fatal outcomes, the median duration from symptom onset to death was 6 days (range 4–10 days), whereas the median time between hospitalization and death was 1 day (range 1–5 days).

**Table:** Patient demographics and outcomes during a Brazilian spotted fever outbreak, Americana, Brazil, 2018*

Age, y/sex	Race or skin color	Onset of symptoms	No. medical consultations before diagnosis	Hospitalization date	Treatment start date†	Clinical outcome	Date of outcome
45/M	White	Apr 26	2	May 7	May 7	Cure	May 21
23/M	White	Apr 29	2	May 3	May 3	Death	May 4
53/M	White	Apr 29	4	May 4	May 5	Death	May 5
7/F	White	Apr 30	3	May 4	May 4	Death	May 4
51/M	Brown	May 01	1	NA	May 2	Cure	NA
65/M	Black	May 04	3	May 13	May 14	Death	May 14
45/M	Brown	May 05	3	May 9	May 9	Death	May 14
6/M	White	May 06	2	May 8	May 9	Cure	May 18
60/M	White	May 06	4	May 8	May 10	Death	May 11
59/M	White	May 07	2	May 11	May 11	Death	May 11
4/M	Brown	May 18	2	May 23	May 24	Death	May 24
37/M	Brown	May 22	1	May 27	May 28	Death	May 28
2/M	White	May 30	3	NA	Jun 3	Death	Jun 3
49/M	White	Jun 02	2	NA	Jun 7	Death	Jun 7
49/M	Brown	Jun 07	1	NA	Jun 9	Cure	NA

*NA, not applicable. †Treatment with doxycycline or chloramphenicol.

Most patients were working-age adults (20–59 years), although the inclusion of children is notable. Previous studies suggest severe manifestations and mortality reaching 80% in children (*5*–*7*). We found that the low frequency of widespread skin rashes (5 of 15 in general, 1 of 6 in patients of color), a clinical clue for BSF suspicion even in cases that already showed signs of severity, might have contributed to late suspicion with a consequent high mortality rate.

Survivors sought care during the first 3 days of symptoms and received doxycycline at median day 3 (range 2–4) compared with day 6 (range 4–9) for nonsurvivors. Time from onset to hospitalization was also shorter among survivors (3 vs. 5 days). We found that early identification of the disease during the prodromal phase, especially when associated with nonspecific clinical symptoms and a history of exposure to tick habitats, ideally by day 3 of symptom onset, can provide an opportunity for immediate outpatient treatment with oral antimicrobial drugs to avoid hospitalization (*4*).

During the investigation, the municipal epidemiologic surveillance team gathered information from patients and family members. Eight patients reported the occurrence of tick bites. All patients had a history of engaging in outdoor leisure activities (e.g., fishing, hiking) <15 days before symptom onset in a waterside forest area located at the confluence of the Atibaia and Jaguari Rivers. An acarological survey that applied a CO_2_ trap technique conducted at 10 sites in this area on June 14, 2018, collected 403 *A. sculptum* ticks (277 larvae, 110 nymphs, and 16 adults) and 82 *A. dubitatum* ticks (30 larvae, 49 nymphs, and 3 adults). We did not test ticks for *R. rickettsii* bacteria.

Unlike what we observed in this outbreak, in endemic areas of BSF where *A. sculptum* ticks are the vector, cases typically concentrate during July– November, when nymphs, the main life stage involved in transmitting *R. rickettsii* to humans, are most abundant in the environment (*2*,*4*,*8*). High infestation by immature stages (larvae and nymphs) of the vector in the environment and high turnover of capybaras susceptible to infection are factors that possibly enabled the occurrence of this outbreak outside the expected epidemiologic pattern, instead during the seasonal peak of larval abundance (*8*).

Even though this region has a long history of BSF endemicity, during this outbreak, the alerts were not timely enough for healthcare professionals in the region. We noted delays in clinical suspicion and diagnosis of BSF in most patients who died, highlighting that BSF remains a relevant public health challenge for health services and that early diagnosis is necessary to avert deaths.

Appendix Additional information about outbreak of Brazilian spotted fever, southeastern Brazil, 2018.
