# Supplementary material for: Outbreak of Brazilian Spotted Fever, Southeastern Brazil, 2018
Source: Emerg Infect Dis. 2026 Sep;32(9):1516–8. doi: 10.3201/eid3209.260548 (PMC13555716; doi:10.3201/eid3209.260548)
Supplement: Supplementary file 1 [file 26-0548-Techapp-s1.pdf]

Article DOI: https://doi.org/10.3201/eid3209.260548

EID cannot ensure accessibility for supplementary materials supplied by authors. Readers who have difficulty accessing supplementary content should contact the authors for assistance.

Outbreak of Brazilian Spotted Fever, Southeastern Brazil, 2018

Appendix

Appendix Table 1. Results of confirmatory laboratory tests for spotted fever group rickettsial infection in patients of the Brazilian spotted fever outbreak, Americana, SP, Brazil, 2018

Age	Sex	Onset of symptoms	Collection 1st sample	Collection 2nd sample	IFA* 1st sample (IgG titer)	IFA 2nd sample (IgG titer)	PCR†
45	Male	April 26	May 07	May 21	<1/64	> = 1/256	
23	Male	April 29	May 03	-	-	-	Detectable
53	Male	April 29	May 05	-	-	-	Detectable
7	Female	April 30	May 04	-	-	-	Detectable
51	Male	May 01	May 01	May 16	<1/64	> = 1/256	
65	Male	May 04	May 14	-	-	-	Detectable
45	Male	May 05	May 09	-	-	-	Detectable
6	Male	May 06	May 09	May 24	<1/64	> = 1/256	
60	Male	May 06	May 10	-	-	-	Detectable
59	Male	May 07	May 11	-	-	-	Detectable
4	Male	May 18	May 24	-	-	-	Detectable
37	Male	May 22	May 28	-	-	-	Detectable
2	Male	May 30	June 03	-	-	-	Detectable
49	Male	June 02	June 08	-	-	-	Detectable
49	Male	June 07	June 09	June 28	<1/64	> = 1/256	

*IFA: Indirect Immunofluorescence Antibody *R. rickettsii* Assay (paired serum samples). Reference value: IgG titer <1/64, convalescent seroconversion IgG > = 1/256.
†PCR: Real-time Polymerase Chain Reaction - gene regions searched: gltA (TaqMan), for *Rickettsia spp.*, internal control of the human RNase P reaction (TaqMan), and a gene encoding hypothetical protein A1G_04230 (GenBank accession no. ABV76353; *R. rickettsii* Sheila Smith strain), for *R. rickettsii*. Method based on the references: Labruna MB et al. J Clin Microbiol. 2004 Jan;42 (*1*):90–8; Kato CY et al. J Clin Microbiol. 2013;51(!):314–7. Recommended for elucidating fatal cases by Ministry of Health Brazil.

Appendix Table 2. Clinical characteristics presented at the time of the suspicion in the patients of the Brazilian spotted fever outbreak, Americana, SP, Brazil, 2018

Age	Sex	Clinical features(at the time of suspicion and notification)	Clinical outcome
45	Male	Fever, headache, myalgia, abdominal pain, diarrhea	Cure
23	Male	Fever, myalgia, abdominal pain, nausea, prostration	Death
53	Male	Fever, headache, myalgia, nausea, prostration, respiratory distress	Death
7	Female	Fever, abdominal pain, nausea, petechiae, respiratory distress, convulsions, hemorrhagic suffusion	Death
51	Male	Fever, headache, myalgia, abdominal pain, diarrhea, prostration	Cure
65	Male	Fever, headache, myalgia, abdominal pain, prostration, jaundice, convulsions	Death
45	Male	Fever, headache, myalgia, rash, respiratory distress, acute kidney injury	Death
6	Male	Fever, headache, nausea, rash	Cure
60	Male	Fever, headache, myalgia, nausea, prostration, rash, jaundice, convulsions, coma, acute kidney injury	Death
59	Male	Fever, headache, myalgia, abdominal pain, nausea, prostration, respiratory distress, jaundice, acute kidney injury, shock	Death
4	Male	Fever, headache, myalgia, abdominal pain, nausea, prostration, diarrhea, jaundice, acute kidney injury. shock	Death
37	Male	Fever, headache, myalgia, nausea	Death
2	Male	Fever, prostration, rash, petechiae, respiratory distress, shock	Death
49	Male	Fever, headache, abdominal pain	Death
49	Male	Fever, headache, myalgia, nausea	Cure

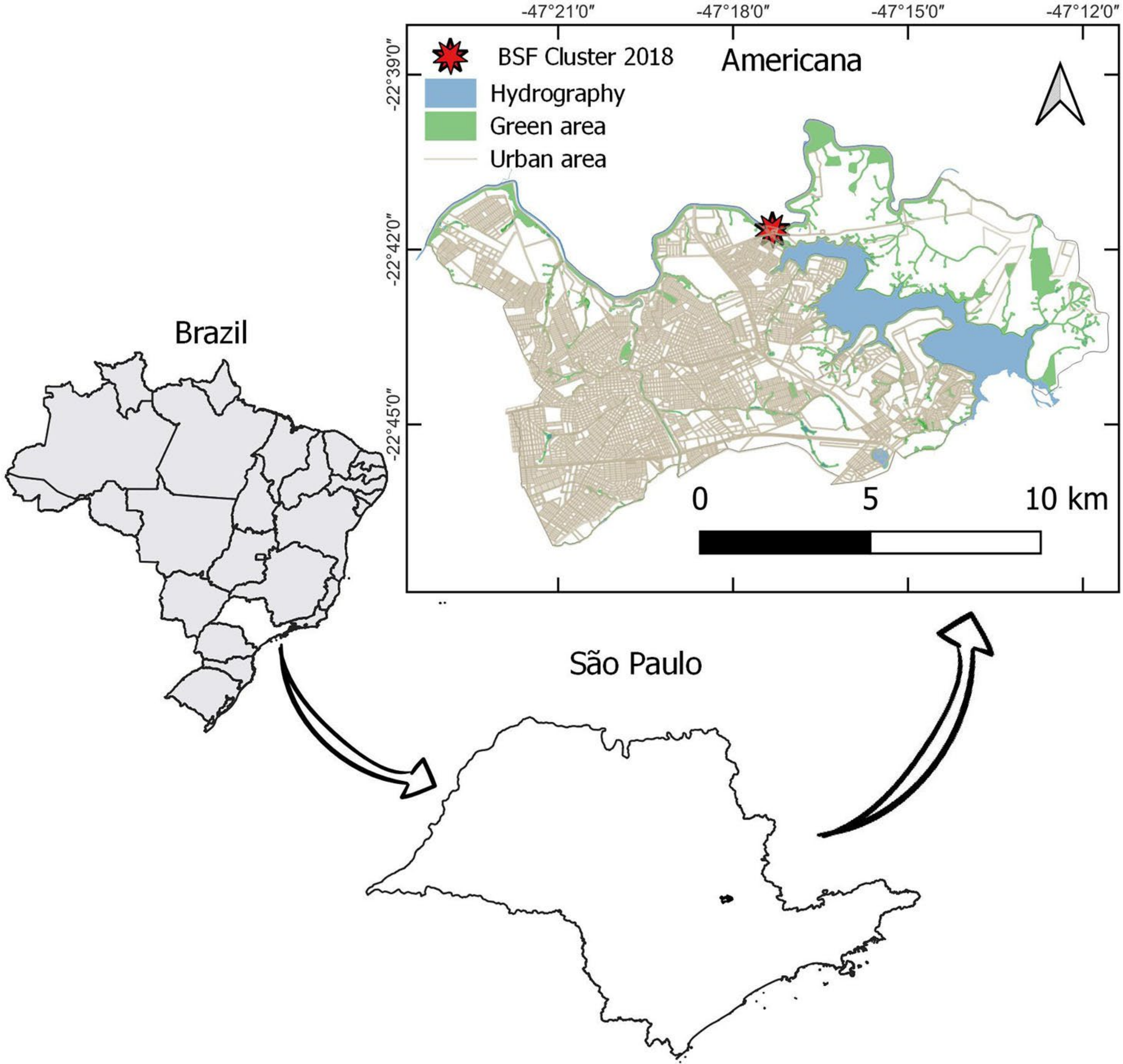

Figure. Map of Brazil, Sao Paulo, and the city of Americana.
